# On Otorrhœa Purulenta; with Cases

**Published:** 1829-10-01

**Authors:** 


### XLVI.

### ON THE OTORRHŒA PURULENTA. By J. BURNE, M.D.

The fourth and fifth Numbers of the

Midland Medical and Surgical Reporter contain a very useful practical paper on Otorrhœa by Dr. Burne. This young physician is already known to the profession by his work on Fever, and his opportunities of cultivating morbid anatomy must be great, from the official situation he holds or held in the Borough hospitals. The paper to which we shall immediately proceed is a long one, but analysis, no doubt, will enable us to curtail it considerably of its fair proportions.

Dr. B. remarks, and we believe with justice, that the disease is in general ill understood; the aurist getting the commencement, when the ear only is affected, the physician the termination, when the cerebrum is implicated, and neither of them seeing or understanding the case as a whole. The otorrhœa, or chronic discharge from the ear, may be mucous or purulent, or mucous originally and purulent afterwards. The otorrhœa mucosa is common in children, confined to the external meatus, and generally yields to treatment, or subsides on the arrival of puberty. The otorrhœa purulenta, on the contrary, is deep-seated and dangerous, and may depend on inflammation of the external ear, the cavity of the tympanum and the bones which it contains, or the internal ear, consisting of the vestibule, semicircular canals, and cochlea. The mucous lining of the tympanum is the part most commonly affected, and the retention of the matter which is formed is the cause of the serious mischief.

"The inflammation of the tympanum, or the otitis interna, manifests itself by a severe pain deep-seated in the ear, which rapidly increases, and affects the whole corresponding side of the head, producing an intense hemicrania. A sympathetic febrile movement is quickly developed; the pain extends itself in the direction of the eustachian tube, and of the mastoid cells, and is exceedingly aggravated by the slightest noise, or the least motion of the head. The pain now acquires a throbbing character; the middle ear, viz. the cavity of the tympanum, is soon filled with a muco-purulent secretion, which having no outlet, in consequence of the canal of the eustachian tube being obstructed by tumefaction, produces a most distressing sensation of tension. To these symptoms, delirium not unfrequently succeeds, and all the sufferings continue without remission until the confined matter has worked itself an outlet."

The matter may discharge itself through a spontaneous perforation of the membrana tympani, by the eustachian tube, or through a fistulous opening in the mastoid process; the first, according to Mons. Itard, the most frequent. When the discharge takes place, it is sudden, copious, and immediately succeeded by a subsidence of the violent symptoms. Sometimes the matter will escape both by the meatus externus and eustachian tube simultaneously, or, what happens oftener, will alternate. The disease is not unfrequently chronic, and produced by the inflammation travelling from the throat along the eustachian tube. This happens especially in the inflammation of the fauces attending eruptive diseases, and hence otorrhœa is frequently dated from an attack of small-pox or scarlet fever.

When the matter lodges in the tympanum, from whatever cause, slow, but continual, insidious, and destructive disorganization ensues, attended only by obscure symptoms, except when accident, as the taking cold, kindles up more urgent and alarming mischief. Life is threatened in these attacks, by one of which the patient is sooner or later cut off.

When the confined matter has worked its way out by ulceration of the membrana tympani, the second stage of the disease is established. The air now getting access to the retained matter, it is decomposed, its irritating qualities are increased, the lining membrane ulcerates, and the bony structure is exposed. The pus being more pent up in the mastoid cells than elsewhere, they are first affected, and may be greatly undermined before we are aware. Little is complained of but an obscure pain in the region of the middle ear, spreading from thence over that side of the head, and accompanied with dulness of hearing. The face is apt to be sweaty, the hair greasy, and, in most cases, the inflammation and thickening extend to the posterior nares, making the voice thick and obliging the patient to breathe through the mouth.

"As the caries advances, the mastoid process becomes tender on pressure, and the integuments about it puffy and vascular; and by and by, the perforation of the bone being completed, the matter escapes, and forms an abscess underneath the integuments. The point where the carious perforation happens, is generally at the anterior part of the groove, from which arises the digastric muscle. The abscess does not burst readily, but extends itself upwards behind the ear, the soft parts offering less resistance in this direction; but there are examples in which the abscess descends under the stylo-mastoideus, and points low down in the neck; it never extends backwards, being prevented by the digastric and trachelo-mastoid muscles, and the deep cervical fascia.

"The caries of the mastoid cells does not confine itself to this outward direction, but spreads on every side, and next affects that part of the posterior surface of the base of the petrous portions of the temporal bone, which forms the fossa for the lateral sinus, and the posterior wall of the mastoid cells; the bone is here naturally thin, and when carious presents a worm-eaten appearance. Through these carious perforations, the matter penetrates, and irritates and detaches the dura mater, and gives rise to more decided cerebral symptoms. The same thing sometimes happens on the superior surface of the petrous portion, in that part which forms the roof of the mastoid cells, but the bone being thicker at this point, is less easily penetrated. Should the patient survive, the caries will go on to ravage the internal ear, viz. the labyrinth: the membranes which close the fenestra ovalis, and the fenestra rotunda, are destroyed, and the small bones of the tympanum discharged through the external meatus; the irritating matter insinuates itself into the vestibule, semicircular canals, and scalæ of the cochlea, which, like the mastoid cells, it disorganizes; and in this way all the sinuosities and cavities, and bony fabric of the ear, are destroyed, and confounded together; and the petrous portion and mastoid process of the temporal bone, present one large carious excavation.

"In the course of this general devastation, is involved the aqueduct of Fallopius, and the portio dura and fascial nerve, the disorganization of which gives rise to particular symptoms, as neuralgic pains, convulsive twitchings, and lastly, paralysis of the muscles of the corresponding side of the face."

The membranes of the brain become affected at an earlier or later period of the caries of the temporal bone, and the patient may be then carried off by a meningitis. At other times the membranes ulcerate, and so does the brain itself, which presents a suppurating cavity more or less deep and extensive. Whilst the disease is confined to the ear, the otorrhœa varies much in quantity, particularly if the eustachian tube is pervious, being at one time profuse by the meatus, at another by the tube. The discharge by the latter collects in the pharynx during sleep, and is hawked up in the morning in considerable quantities. When the discharge is suppressed, as from cold, the pain in the ear and head is much increased, and there is symptomatic fever, attacks of which nature are frequent. If the labyrinth becomes affected, the patient's sufferings are excruciating, but the disorganization frequently goes on to affect the brain and destroy the patient, without this implication taking place.

When the membranes of the brain become affected, the pain, of course, is no longer referred to the ear; there is a deep-seated severe throbbing pain in the middle of one side of the head, with great tenderness of the scalp on the same side, so that the patient cannot lie upon it; there is no marked suffusion of the face, nor vividness of the eye, and although there is delirium, it is little active, and is complicated with a heaviness of the head and some stupor; the pulse is rather tight than full, and sharp than strong; the symptomatic fever moderate in the day, but offering a marked exacerbation at night. It is from the symptoms above enumerated, coupled, of course, with the previous history of the case, that the practitioner is to distinguish this secondary meningitis from the idiopathic form of the disease, a distinction of some importance in regard to the employment of depletion.

*Treatment.* When a puriform discharge from the tympanum is once established, our object should be to prevent its accumulation by injections. When the membrana tympani is perforated by ulceration, they can easily be thrown in by the meatus externus, but the most formidable obstacle to a cure is the lodgment of matter in the mastoid cells. The only measure which presents itself in such a case is to perforate the mastoid process, and inject by the artificial opening, a plan recommended highly by the younger Sprengel in his History of Surgery. Should the membrana tympani not have been perforated by ulceration, and the discharge have taken place only by the eustachian tube, this method would offer equal advantages, the injections passing directly from the cells through the tympanum, and down the eustachian tube into the pharynx. We have never seen this operation performed, but the excellent effects of removing portions of the external table of the cranial bones, for lodgements of matter in the diploë, would lead us to entertain a very favourable opinion of the proposal. The trephine is the best instrument to employ on such an occasion. For the composition of the injections, Dr. Burne believes that warm milk and water will answer as well any thing, but M. Itard recommends astringents, especially the potassa fusa dissolved in rose water. We should think that the dilute solutions of the sulphate of zinc, or nitrate of silver, or some other of the washes commonly employed in the treatment of ulcers depending on carious bone, might be useful.

Care must be taken to ascertain that the caries has not penetrated the skull before we have recourse to the injections, as the consequences in such a case might be serious. The occasional attacks of inflammation in the ear soon yield to mild antiphlogistic treatment, and the application of a few leeches to the mastoid process. When, however, the mischief has spread to the brain, a return of the otorrhœal discharge must be sedulously courted by fomentations and poultices to the side of the head, and bloodletting, so far as to keep the inflammation within limits consistent with life. If drawn too freely, it cannot put an end to the attack, the cause still operating, but as soon as the discharge returns, the patient will sink.

"In every attack of this nature, the practitioner should examine the affected side of the head attentively, as an attack is apt to supervene at the moment of the formation of abscess behind the ear, from the carious perforation of the mastoid process; in this case a free incision is to be made into the fluctuating tumor, and the matter allowed to discharge itself, which will at once alleviate the sufferings, and give a favourable turn to the symptoms, provided discretion has been observed in the abstraction of blood."

Such are our author's principles of treatment, and we own that we are surprised at his almost total silence respecting local depletion and counter-irritating measures. In the earlier stage, that previous to ulceration, he does not even hint at them, and afterwards he merely recommends a few leeches and "mild antiphlogistic treatment" during the temporary accesses of inflammation. We would certainly advise the more frequent application of leeches, blisters behind the ear, the antimonial ointment, or cupping, according to circumstances. When the disease is become very chronic, and the discharge is copious, leeches and cupping are not required, but still we would not altogether abandon counter-irritation by the tartar-emetic. We shall now advert to the cases appended to the Doctor's paper, and intended to illustrate his precepts and practice.

*Case* 1. CHRONIC PURIFORM DISCHARGE BY THE MEATUS EXTERNUS AND EUSTACHIAN TUBE.

Elizabeth Waugh, æt. 15, is constantly subject to an offensive discharge from the throat, which collects during sleep, and is hawked up in considerable quantity in the morning. A discharge of the same kind issues sometimes from the left ear. She has deep-seated extensive pain in the head between the ears; dull aching pain in the left, occasionally of a pricking character; sometimes she is deaf in this ear, at others, the hearing partly returns. She has frequent attacks of violent pain in the part, with severe head-ache, fever, and occasionally vomiting, which are always preceded by a suppression of the discharge, attributed to cold. The nares are pervious, but small, which obliges her to keep her mouth constantly open; the upper eye-lids droop; the face is pale and sallow; and she suffers from excessive constipation of the bowels, passing a fortnight without a dejection. She began to menstruate in the Spring of last year, and has continued to do so *irregularly*.

In a consultation with Mr. Briggs, the disease was not considered far enough advanced to justify perforation of the mastoid process, a determination in which the consultants, we think, acted wisely. Injections, leeches during the attacks, and daily evacuation of the bowels were attended with excellent effects. The disease, however, is still going on.

All who are conversant with the maladies of the female sex, will not fail to recognize a great deal of nervous affection mixed up with the *aural* disease in the foregoing case. The irregular menstruation, constipated bowels, and pain over almost the whole of the head, are characteristic features of those complaints, which attack, in a greater or less degree, the great majority of young unmarried females. Attention to the bowels is a leading point, and Dr. Burne particularly adverts to its excellent effects in the present instance.

*Case* 2. Otorrhœa—Abscess behind the Right Ear—Head Symptoms—Case mistaken for Idiopathic Phrenitis—Death.

"The patient was a young lady about 25 years of age, and had been ill already ten days, when I was desired to see her in consultation. She was supposed to be labouring under an idiopathic phrenitis, and had been bled copiously and repeatedly. The attack commenced with head-ache and bilious vomiting.

"The prominent complaints were a violent throbbing pain in the head, with tenderness of the scalp, on the right side, which made her unwilling to lie upon it; great pain also in the middle of the arms. She had a pale face and a vacant look; the pupils were dilated, and light thrown upon the eyes made them ache; when the eyes were closed she was disturbed by frightful images, which entirely prevented sleep; the tongue was white, and at times she had been sick, and had vomited; the pulse numbered 140, was compressible, and the artery yielding to the impulse of the heart, and she had profuse perspirations. There was tenderness in the præcordia, and the bowels were not satisfactorily open; at this time she was sensible, but delirium, unaccompanied with restlessness, prevailed through the night.

"The case appeared to me exceedingly ambiguous; it was evidently not an idiopathic phrenitis, and as there was much tenderness in the præcordia with a confined state of the bowels, I thought it possible the condition of the head might depend on deranged function of some of the abdominal viscera, and therefore that a correct diagnosis could not be formed until the bowels had been properly evacuated. To this end aperients were prescribed, which produced on the next day some slight general relief; but all the head symptoms continued.

"Being still undecided as to the nature of the complaint, I proceeded to examine the head, and discovered a large abscess behind the right ear, covering the mastoid process and inferior angle of the parietal bone. All doubt was now cleared up, and on enquiry, I found she had been subject to pain in that ear, and also to purulent discharge from it, so that it was evident the disease was an inflammation, supervening on otorrhœa at the period of the carious perforation of the mastoid process, and formation of abscess behind the ear. The abscess was opened by the surgeon, and discharged a small tea-cup full of most offensive matter, indicating disease of the bony structure; she was directed to take tonics, and an anodyne at bed-time.

"On the following day, she expressed herself as having passed a more comfortable night, and as suffering much less pain in the head; but notwithstanding this mitigation of suffering, it was too visible that the powers of life were fast declining. She had now the aspect of a woman exhausted by uterine hæmorrhage, the face being a pale sallow, exsangueous and rather bloated; the conjunctiva blanched, and the eyes glassy; the pulse had decreased in frequency, from 140 to 128, but its stroke was of a mock fulness, short and unsteady, and very compressible. After this, the delirium became constant, the respiration grew hurried, the circulation feeble, and she expired at the end of two days. Inspection of the body was not permitted."

Dr. Burne makes no remark on the foregoing case, no doubt from motives of delicacy and personal considerations. Can it be doubted that the poor lady died from the injudicious treatment adopted, we mean from the copious and repeated bleedings for idiopathic phrenitis? Alas, the aspect of *uterine hæmorrhage* bespeaks but too truly the victim offered up at the Moloch shrine of inflammation.

*Case* 3. Otorrhœa — Cranial Implication—Death—Dissection.

Mr. ———, æt. 40, a patient of Mr. Finch, of Greenwich, had been subject to puriform discharge from the left ear for upwards of 15 years, during the latter part of which time he suffered intense pain in the head, particularly on the affected side. When, from accident or otherwise, the discharge was checked, there supervened inflammation of the membranes of the brain, which always placed his life in danger and eventually destroyed him. During the course of the disease, there took place paralysis of the muscles supplied by the left portio dura, and, a short time before death, an abscess, attended with severe cerebral symptoms, shewed itself behind the ear, at the base of the mastoid process. This was opened by Mr. Callaway, a quantity of ill-conditioned matter let out, and the bones of the mastoid cells discovered to be diseased. A number of medical men were consulted, no two of whom would seem to have agreed, no one of whom would seem to have been of use. An aurist arrested the discharge and increased the disease; another, we suppose a dentist, discovered disease of the antrum, and proposed the extraction of a tooth; in short, the case was very little understood, the patient died, and the following is the account of the dissection.

"*Sectio Cadaveris*, 48 *hours after death*. The body emaciated to the *second degree*,* having been corpulent.

"The dura mater visible on the removal of the upper part of the cranium, was sound, but blood oozed very freely from the torn vessels. The right hemisphere presented considerable arborescent vascularity of the pia mater, with some serosity in the furrows of the convolutions; but the veins between the convolutions not larger than is natural. At the fore and inner part of the anterior lobe of this hemisphere, close to the falx, was some yellow consistent albumen in one of the furrows of the convolutions, and exactly opposite to it was a similar effusion, in the anterior lobe of the left hemisphere. Portions of consistent albuminous effusion were also seen under the arachnoid, in the left temporal fossa in the fore part of the middle lobe of the left hemisphere, in the tractus opticus, on each side of the tuber annulare, and on the inferior surface of the right lobe of the cerebellum. The left hemisphere presented an arborescent vascularity greater than the right, and the veins between the convolutions were larger and fuller.

* What this *second* degree of emaciation may be, we really do by no means profess to know. Perhaps they keep a *gamut*, or scale of leanness in the Borough!—*Rev*.

"The substance of the brain was preternaturally firm, especially the left hemisphere, which was firmer than the right. In the medullary substance the bloody points were very numerous, about the junction of the middle and posterior lobes; and in the centrum ovale, the vessels in the medullary substance were remarkably large, and conspicuously so on the left side.

"In the ventricles the veins were of an unusual size, and there was a general duskiness of the corpora striata and thalami; the commissura mollis was wanting, the thalami being in imperfect apposition; and, at the anterior and upper part of each thalamus, was a tuberosity, softer on the left than on the right side.

"The left lobe of the cerebellum was soft, though not entirely disorganized at its anterior inferior part opposite the internal auditory foramen; the convolutions at this part were unattached to each other, and the pia mater was beginning to ulcerate. At the base of the skull, in the neighbourhood of the internal auditory foramen, was a fœtid sero-purulent effusion, to the extent of six drachms, part of which flowed in the direction of the vertebral canal.

"The dura mater, at the base of the skull, was sound on its cerebral surface, except that it was of a darker colour over the petrous portion of the left temporal bone, than of the right.

"The diseased temporal bone was taken out, and when the soft parts had been removed by maceration, it afforded a fine specimen of the carious disorganization which accompanied the chronic puriform discharge from the ear. The disorganization had entirely broken down the mastoid cells, converted them into one large excavation, extending in the direction of the mastoid process, which was perforated at the root on the inside, the point where the abscess formed. The caries had also enlarged very much the natural opening between the cells and the tympanum, and had involved the aqueduct of Fallopius; it had also penetrated the vestibule and cochlea, thus forming a communication with the internal auditory foramen, by which the matter had found its way to the base of the skull, as above described. The roof and posterior wall of the mastoid cells were perforated by the carious disorganization, by a number of small holes, particularly the posterior wall, which forms the fossa for the lateral sinus, so as to give this part of the bone a worm-eaten appearance; and through these foramina, matter had passed and burrowed between the dura mater and the cranium."

We think that the foregoing account is in many parts needlessly minute, and may fall under the bitter sarcasm, levelled by John Bell at certain anatomical descriptions. However, it is better to sin this way, than by laxity and negligence, the besetting sin of most pathological writings in this country.

*Case* 4. Otorrhœa — Caries — Extensive Disorganization of the Brain.

"Harriet Ranyam, aged 19 years, of delicate health from infancy. Eight years ago she became affected with purulent discharge from the external meatus of the right ear, which was accompanied with severe pain on the corresponding side of the head, and about the occiput. The pain was very much aggravated when, from any cause, the discharge was diminished, and particularly when it ceased, which happened not unfrequently.

"Dr. Wilmot saw her for the first time on the 29th September; she then laboured under a distracting pain on the right side of the head, about the occiput; the habitual discharge from the ear had ceased suddenly; the pulse was feeble and oppressed, and the tongue white and loaded; the stomach was irritable, and ejected a greenish fluid; the aspect of the face was jaundiced, and the strength much exhausted.

"Next day the bowels acted freely, and the discharge from the ear returned to a profuse degree; she was in a state of high excitement, apparently from re-action. Leeches were directed to be applied to the temples and behind the ears. From this period she grew worse, and, on the 5th day, the pulse exceeded 120, and the skin was clammy. Leeches were again ordered to the temples, during the application of which she turned upon the left side and expired.

"*Sectio Cadaveris.*—The dura mater was found to be detached from the petrous portion of the temporal bone, and on the posterior face were several small carious perforations which communicated with the mastoid cells and tympanum. The middle lobe of the right hemisphere of the brain was extensively destroyed by ulceration, and presented an excavation, like abscess, which contained about one ounce of offensive pus; the walls of this excavation were very dark and sloughy, and the surrounding medullary and cineritious substance softened.

"The temporal bone being removed for the further examination of the ear, it was discovered that the membrana tympani had been destroyed, and the incus and os orbiculare carried away by the suppuration, the malleus and stapes still remaining. The tympanum and mastoid cells formed one carious excavation, which communicated with the vestibule and semi-circular canals. The cochlea appeared sound."

This closes the paper, and our analysis. We return Dr. B. many thanks for the instruction it conveys, and we hope that our readers will profit by its perusal as well as ourselves.

---

